# Medical needs during the Kumamoto heavy rain 2020: analysis from emergency medical teams’ responses

**DOI:** 10.1186/s12873-024-01009-7

**Published:** 2024-05-31

**Authors:** Akihiro Taji, Yui Yumiya, Odgerel Chimed-Ochir, Ami Fukunaga, Yoko Tsurugi, Koji Kiwaki, Kouki Akahoshi, Yoshiki Toyokuni, Kayako Chishima, Seiji Mimura, Akinori Wakai, Hisayoshi Kondo, Yuichi Koido, Tatsuhiko Kubo

**Affiliations:** 1 Department of Public Health and Health Policy Graduate School of Biomedical and Health Sciences, Hiroshima University, 1-2-3 Kasumi, Minami-ku, Hiroshima, 734-0037 Japan; 2 Aso Public Health Center, Kumamoto Prefecture Government, 2402 Miyaji, Ichinomiya-machi, Aso City, Kumamoto, 869-2612 Japan; 3 Hitoyoshi Public Health Center, Kumamoto Prefectural Government, 86-1 Nishiaidashimo-machi, Hitoyoshi-city, Kumamoto, 868-8503 Japan; 4 National Hospital Organization Headquarters DMAT Secretariat MHLW Japan, 3256 Midori-cho Tachikawa, Tokyo, 190-8579 Japan; 5 National Hospital Organization Headquarters DMAT Secretariat MHLW Japan, 2-1-14 Houenzaka Chuo-ku, Osaka City, Osaka 540-0006 Japan

**Keywords:** Emergency medical team, J-SPEED, Emergency medical team minimum data set, Disaster, Kumamoto heavy rain, Japan

## Abstract

**Background:**

Rainfall-induced floods represented 70% of the disasters in Japan from 1985 to 2018 and caused various health problems. To improve preparedness and preventive measures, more information is needed on the health problems caused by heavy rain. However, it has proven challenging to collect health data surrounding disasters due to various inhibiting factors such as environmental hazards and logistical constraints. In response to the Kumamoto Heavy Rain 2020, Emergency Medical Teams (EMTs) used J-SPEED (Japan-Surveillance in Post Extreme Emergencies and Disasters) as a daily reporting tool, collecting patient data and sending it to an EMTCC (EMT Coordination Cell) during the response. We performed a descriptive epidemiological analysis using J-SPEED data to better understand the health problems arising from the Kumamoto Heavy Rain 2020 in Japan.

**Methods:**

During the Kumamoto Heavy Rain 2020 from July 5 to July 31, 2020, 79 EMTs used the J-SPEED form to submit daily reports to the EMTCC on the number and types of health problems they treated. We analyzed the 207 daily reports, categorizing the data by age, gender, and time period.

**Results:**

Among the 816 reported consultations, women accounted for 51% and men accounted for 49%. The majority of patients were elderly (62.1%), followed by adults (32.8%), and children (5%). The most common health issues included treatment interruption (12.4%), hypertension (12.0%), wounds (10.8%), minor trauma (9.6%), and disaster-related stress symptoms (7.4%). Consultations followed six phases during the disaster response, with the highest occurrence during the hyperacute and acute phases. Directly disaster-related events comprised 13.9% of consultations, indirectly related events comprised 52.0%, and unrelated events comprised 34.0%. As the response phases progressed, the proportions of directly and indirectly related events decreased while that of unrelated events increased.

**Conclusion:**

By harnessing data captured by J-SPEED, this research demonstrates the feasibility of collecting, quantifying, and analyzing data using a uniform format. Comparison of the present findings with those of two previous analyses of J-SPEED data from other disaster scenarios that varied in time, location, and/or disaster type showcases the potential to use analysis of past experiences to advancing knowledge on disaster medicine and disaster public health.

**Supplementary Information:**

The online version contains supplementary material available at 10.1186/s12873-024-01009-7.

## Background

Due to the effects of global warming-driven climate change, the world is witnessing a noticeable uptick in the frequency of natural disasters such as floods, typhoons, and hurricanes [1]. In Japan, floods have emerged as the predominant natural disaster, accounting for approximately 70% of the natural disasters documented from 1985 to 2018 [2]. The period spanning from 2012 to 2021 witnessed twice the annual occurrences of heavy [2]exceeding 400 mm per day when compared to the period of 1976–1985 [3].

It is well-documented that floods yield both direct and indirect health damages, including trauma, infectious diseases (e.g., respiratory and gastrointestinal infections), skin diseases, animal and insect bites, mental health problems, and exacerbation of pre-existing medical conditions [4–6]. During the 2018 West Japan Heavy Rain skin diseases emerged as the most prevalent health event, followed by wounds and disaster-related stress symptoms [7]. However, the multiple difficulties limiting the collection of comprehensive data during a disaster, including the busy schedules of disaster responders, logistical hurdles (e.g., those related to communication and road conditions), and complexities in securing consent from disaster [8–10], meant that the quantitative evidence remained somewhat constrained.

During a disaster, Japan deploys emergency medical teams (EMTs) comprising doctors, nurses, logisticians, and coordinators to deliver necessary medical assistance to those in need. These EMTs operate as various entities, including Disaster Medical Assistance Teams, Disaster Assistance Psychiatric Teams, Japan Medical Assistance Teams, and Japanese Red Cross Relief Teams; they are dispatched when requested by governments of the affected localities. EMTs are deployed under the auspices of their respective organizations, which may include private companies and the National Hospital Organization [11].

In Japan, all EMTs currently utilize a standardized data reporting system known as J-SPEED (Japan-Surveillance in Post Extreme Emergencies and Disasters) to provide daily reports on the health data of patient visits to the EMT Coordination Cell (EMTCC). J-SPEED is a standard EMT daily reporting format introduced by the “Joint Committee on Medical Care Records During Disaster”; it was first activated during the Kumamoto Earthquake 2016. The data items of J-SPEED align with the EMT Minimum Data Set (MDS) daily reporting form developed by the World Health Organization [12, 13].

The heavy rain of July 5 to July 31, 2020 (hereinafter referred to as “Kumamoto heavy rain”), resulted in extensive human suffering and property damage in Kumamoto Prefecture and 42 other prefectures. There were 163 human casualties: 84 dead, two missing, 23 seriously injured, and 54 slightly injured. A total of 16,599 homes were damaged: 1,621 were totally destroyed; 4,504 were partially destroyed; 3,503 were partially damaged; 1,681 had above-floor-level flooding; and 5,290 experienced below-floor-level flooding [14]. In areas affected by the Kumamoto heavy rain, J-SPEED was put into operation by the EMTs, allowing for real-time data collection of patient numbers and health-related events. This study analyzes the medical conditions and needs reported by EMTs during the Kumamoto heavy rain. This is crucial for future disaster preparedness to gain insight into the health events that transpired during the flooding.

## Methods

### Study design

This is a descriptive analysis of daily reports collected by EMTs using J-SPEED during the Kumamoto heavy rain that occurred from July 5 to July 31, 2020.

### Data collection

Data were collected using the “J-SPEED+” smartphone application, which has 57 pre-defined items consisting of demographic information, health events, procedure/outcomes, and special notes. As EMTCC advised that heatstroke be included as an additional item, based on the disaster context, a total of 58 items were reported for the Kumamoto heavy rain (see Additional file 1).

The J-SPEED form was used by 79 EMTs to provide daily reports on the number and types of health events to the EMTCC. A total of 816 patient consultations (including follow-up consultations) were recorded in 207 daily medical reports during the Kumamoto heavy rain, from July 5 to July 31, 2020 (Estimated that about 0.4% of the population of the affected areas in Kumamoto Prefecture where human casualties and damage to residential properties were reported (*n* = 204,689 as of July 1, 2020) received consultations [15, 16]).

### Data analysis

All items reported to J-SPEED + were analyzed by age, sex, health events, and disaster phase. J-SPEED + categorized age as 0–14 years old (children), 15–64 years old (adults), and 65 years and older (elders). Women adults were categorized pregnant and non-pregnant.

To identify the disaster phase, a Joinpoint regression model was used. This tool aims to provide models that can best summarize data behaviors or trends [17]. The generated model identifies significant points in a trend and estimates the daily percentage change (DPC), which provides information on the average rate of change between two adjoining points. This analysis was used to identify informative joinpoints at which the patient consultation numbers changed (taken as phase boundaries) during the response period. A more detailed mathematical explanation can be found elsewhere [18]. Based on the five identified joinpoints, the disaster response period was divided into the following: hyperacute phase (response days 1–3), acute phase (response days 4–9), subacute I phase (response days 10–12), subacute II phase (response days 13–17), chronic I phase (response day 18–21), and chronic II phase (response days 22–27). The Cochran–Armitage test for trend was applied to the proportions of patient consultations in each identified phase [19].

Microsoft Excel (Microsoft Corp., Redmond, Washington, USA), STATA v15.1 (STATA Corp; College Station, Texas USA), and Joinpoint Trend Analysis Software V5.0 (National Cancer Institute, Bethesda, Maryland, USA) were used for the analyses.

## Results

Table 1 presents the distribution of consultations by sex and age. Of the total 816 consultations, men constituted 49.0% (*n* = 400) and women accounted for 51.0% (*n* = 416). Regarding age, elderly patients accounted for the highest proportion at 62.1% of all consultations, followed by adults at 32.8%, and children at 5.0%. For gender within the age groups, male patients outnumbered female patients in consultations for children (63.4% vs. 36.6%) and adults (51.1% vs. 48.9%), whereas female patients outnumbered males in consultations for the elderly (46.7% vs. 53.3%).

**Table 1:** Background of patient consultation during the Kumamoto heavy rain 2020

Age	Sex									Total	
	Male			Female Non-pregnant			Female Pregnant				
0–14	26	[63.4%]		15	[36.6%]		0	[0.0%]		41	[100.0%]
	6.5%			3.7%			0.0%			5.0%	
15–64	137	[51.1%]		121	[45.1%]		10	[3.7%]		268	[100.0%]
	34.3%			29.8%			100.0%			32.8%	
65+	237	[46.7%]		270	[53.3%]		0	[0.0%]		507	[100.0%]
	59.3%			66.5%			0.0%			62.1%	
Total	400	[49.0%]		406	[49.8%]		10	[1.2%]		816	[100.0%]
	100.0%			100.0%			100.0%			100.0%	

% in square bracket is percentage of consultations of each sex in each age group (Row percentage)

% without square bracket refers to percentage of consultations of each age group in each sex (Column percentage)

Table 2 depicts the frequency and percentage of the studied health events categorized by age group. Among the total of 816 consultations, the most prevalent five health events were treatment interruption (*n* = 101, 12.4%), hypertension (*n* = 98, 12.0%), wounds (*n* = 88, 10.8%), minor trauma (*n* = 78, 9.6%), and disaster stress-related symptoms (*n* = 60, 7.4%). Regarding the age groups, children most commonly presented with skin disease (*n* = 8, 19.5%), followed by treatment interruption (*n* = 6, 14.6%) and acute respiratory infection (ARI) (*n* = 5, 12.2%). Among adults, wounds (*n* = 35, 13.1%) were the most common, followed by minor injuries (*n* = 30, 11.2%) and disaster stress-related symptoms (*n* = 25, 9.3%). Among the elderly, hypertension emerged as the most prevalent symptom (*n* = 81, 16.0%), followed by treatment interruption (*n* = 78, 15.4%) and wounds (*n* = 50, 9.9%).

**Table 2:** Health events treated at EMTs during the Kumamoto heavy rain 2020

Total number of consultations	0–14 years old			15–64 years old			65 + years old			Total	
	*N*.	%		*N*.	%		*N*.	%		*N*.	%
	41	[5.0%]		268	[32.8%]		507	[62.1%]		816	[100%]
**Health Events**											
Interruption of treatment	6	**14.6%**		17	6.3%		78	**15.4%**		101	12.4%
High blood pressure	1	2.4%		16	6.0%		81	**16.0%**		98	12.0%
Wound	3	7.3%		35	**13.1%**		50	**9.9%**		88	10.8%
Minor trauma (can be treated only by outpatient treatment)	1	2.4%		30	**11.2%**		47	9.3%		78	9.6%
Symptoms related to disaster stress	1	2.4%		25	**9.3%**		34	6.7%		60	7.4%
Skin diseases (other than trauma and burns)	8	**19.5%**		17	6.3%		28	5.5%		53	6.5%
Fever	3	7.3%		11	4.1%		13	2.6%		27	3.3%
Deep vein thrombosis/Suspected pulmonary/cerebral/coronary embolism	0	0.0%		6	2.2%		14	2.8%		20	2.5%
Urgent non-infectious medical needs	0	0.0%		4	1.5%		11	2.2%		15	1.8%
Acute respiratory infection	5	**12.2%**		2	0.7%		3	0.6%		10	1.2%
Burns	1	2.4%		1	0.4%		7	1.4%		9	1.1%
Urgent mental care needs	0	0.0%		3	1.1%		5	1.0%		8	1.0%
Heatstroke	0	0.0%		2	0.7%		6	1.2%		8	1.0%
Gastrointestinal infections, food poisoning	0	0.0%		4	1.5%		3	0.6%		7	0.9%
Fracture	0	0.0%		2	0.7%		4	0.8%		6	0.7%
Bronchial asthma attack	0	0.0%		0	0.0%		4	0.8%		4	0.5%
Moderate trauma (PAT^1^-except red, hospitalization required)	0	0.0%		0	0.0%		4	0.8%		4	0.5%
Artificial dialysis needs	0	0.0%		0	0.0%		2	0.4%		2	0.2%
Severe trunk trauma (PAT^1^-red)	0	0.0%		1	0.4%		0	0.0%		1	0.1%
Suspected tetanus	0	0.0%		0	0.0%		1	0.2%		1	0.1%
Emergency care/nursing care needs	0	0.0%		0	0.0%		1	0.2%		1	0.1%
Urgent drinking water and food needs	0	0.0%		0	0.0%		1	0.2%		1	0.1%
**Other diseases not listed above**	9	22.0%		48	17.9%		87	17.2%		144	17.6%

[%] - Percentage of consultation for each age group

% - Percentage of health events among total consultation

Health events of some patients were not recorded; therefore, total health events are fewer than number of consultation

Following health events with zero cases are not listed in the table: severe head and neck/spine trauma, severe limb trauma, drowning, crash syndrome, suspected measles, acute bloody diarrhea, urgent infectious disease response needs, urgent non-traumatic surgical needs, urgent nutritional support needs, and urgent obstetric support needs

^1^ Physiological and Anatomical Triage method

PAT is used for secondary triage to determine the priority of treatment for patients who require medical treatment based on physiological and/or anatomical factors. (Red: immediate; Yellow: urgent; Green: minor)

**Fig. 1:**
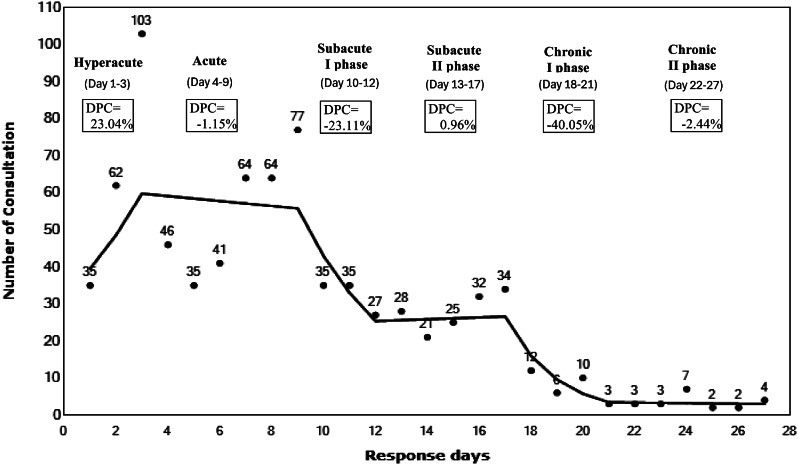
Time-based phase of Kumamoto heavy rain 2020

Figure 1 shows the trend in the total number of patient consultations, as identified by joinpoint regression analysis. For the 27 response days, this analysis identified six phases marked by five distinct change points in the trend: (i) During the initial hyperacute phase (response days 1–3), there was a substantial increase in patient consultations with a DPC of 23.04%. (ii) During the acute phase (response days 4–9), there was a slight decrease in consultations, with a DPC of -1.15%. (iii) During the subacute I phase (response days 10–12), the trend rebounded and swung upward, with a DPC of 23.11%. (iv) During the subacute II phase (response days 13–17), the consultations stabilized to exhibit a modest increase, with a DPC of 0.96%. (v) During the chronic I phase (response days 18–21), patient consultations showed a significant downturn, with a DPC of -40.05%. (vi) Finally, during the chronic II phase (response days 22–27), the number of consultations declined at a slower pace, with a DPC of -2.44%.

**Fig. 2:**
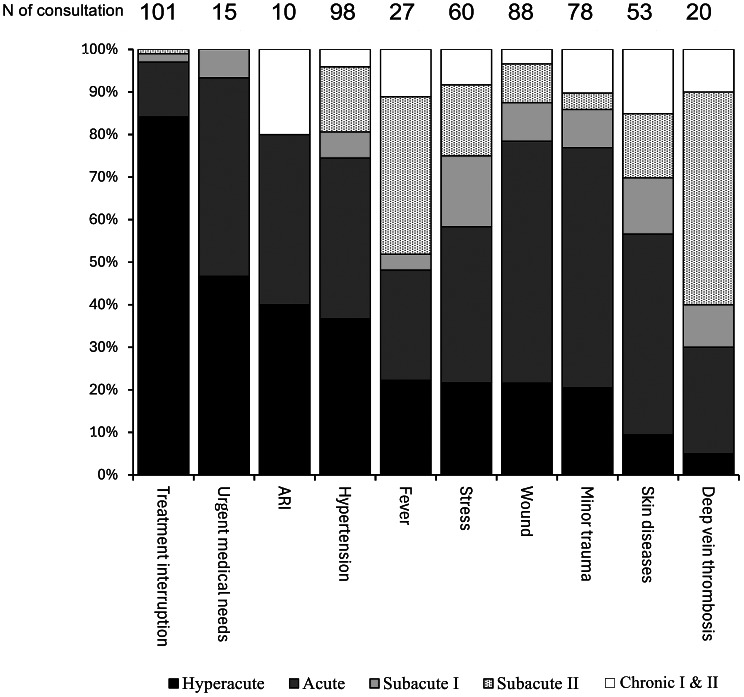
Phase distribution of health events during Kumamoto heavy rain 2020

Figure 2 presents the phase distribution for health events with ten or more cases. This analysis shows that 84.2% of patients experiencing treatment interruption, 46.7% with urgent medical needs, and 40.0% suffering from ARIs visited EMTs during the hyperacute phase of the disaster response. In contrast, the majority of patients with deep vein thrombosis, skin diseases, and minor trauma arrived at EMTs after the hyperacute phase of the disaster response.

**Fig. 3:**
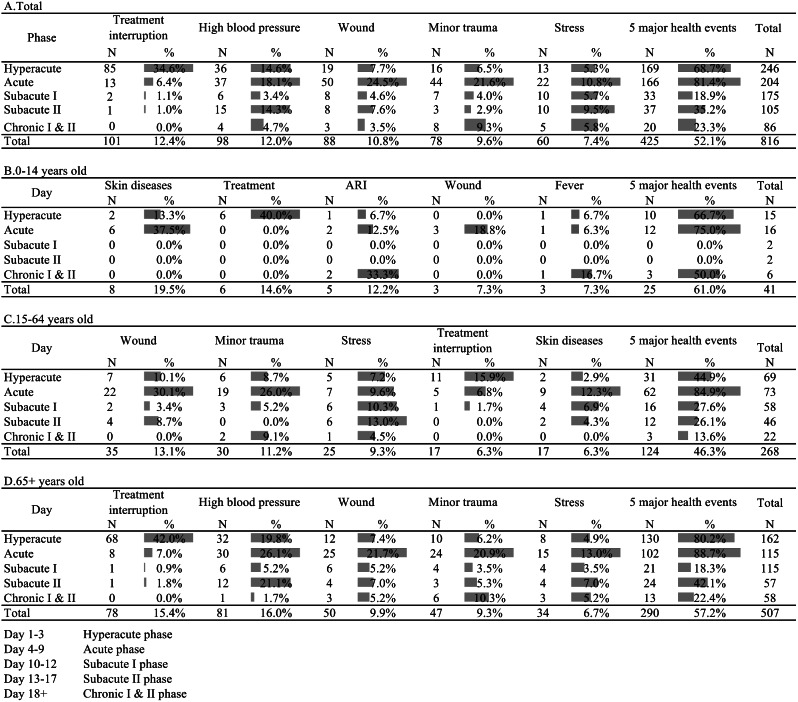
Distribution of top five health events by disaster phase

Figure 3 illustrates the distribution of five major health events by disaster response phase and age group.

Across all patients, the top five major health events were treatment interruptions, hypertension, wounds, minor trauma, and stress; these events collectively accounted for 52.1% of all consultations. Treatment interruptions peaked during the hyperacute phase (*n* = 85, 34.6%) and rapidly decreased thereafter. Wounds (*n* = 50, 24.5%) and minor trauma (*n* = 44, 21.6%) were more prevalent during the acute phase. Similar trends were observed for the adult and elderly age groups.

For children, the most frequent health events were skin diseases, treatment interruption, ARIs, wounds, and fever, which collectively made up 61.0% of the consultations in this group. Among adults, the prevalent health events included wounds, minor trauma, stress, treatment interruptions, and skin diseases, which collectively comprised 46.3% of all consultations. In elderly patients, the top five major health events were treatment interruptions, hypertension, wounds, minor trauma, and stress, which together comprised 57.2% of all consultations.

**Fig. 4:**
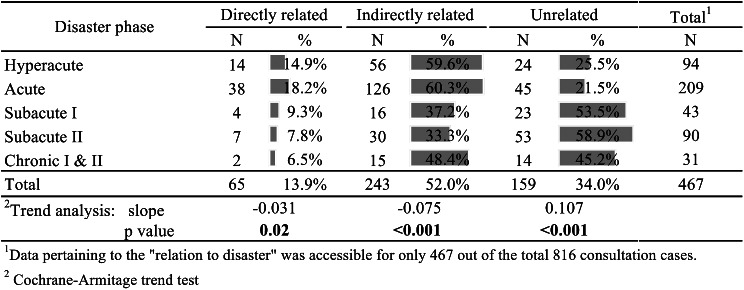
Patient consultation in terms of relation to disaster

Figure 4 shows the breakdown of patient consultations based on their relationship to the disaster. Consultations were categorized as being directly related, indirectly related, or unrelated to the disaster. Directly related, indirectly related, and unrelated events accounted for 13.9%, 52.0%, and 34.0% of the consultations, respectively. The percentage of directly related events (b=-0.031, *p* = 0.02) and indirectly related events (b=-0.075, *p* < 0.001) decreased significantly as the disaster phase progressed, whereas the proportion of unrelated events increased significantly (b = 0.107, *p* < 0.001) as the disaster phase progressed.

## Discussion

From July 5 to July 31, 2020, a total of 816 medical consultations were provided by EMTs. These consultations covered a range of health events, with the top five being treatment interruptions, hypertension, wounds, minor trauma, and symptoms related to disaster stress. These five major occurrences collectively constituted 52.1% of the total consultations. An analysis of these consultations by age group revealed distinct tendencies. Skin diseases, treatment interruptions, and ARIs were more common among children (61.0% of all consultations), whereas wounds, minor traumas, and stress-related symptoms were more common among adults (46.3% of all consultations).

Individuals aged 65 years and older accounted for 62.1% of all consultations. This substantial influx of elderly individuals seeking medical attention can be partly attributed to the aging of Japan’s population, as 28.6% of the Japanese population falls within this age category. The high proportion of elderly is particularly pronounced in regions affected by the Kumamoto heavy rains. For example, Hitoyoshi City and Kuma Village, which were directly affected by the Kumamoto heavy rain, have elderly proportions of 37.9% and 44.8%, respectively [20]. Furthermore, the Sendai Framework for Disaster Risk Reduction 2015–2030 emphasizes that vulnerable populations, including the elderly, women, individuals with disabilities, and children, are disproportionately affected by disasters [21]. This could also contribute to explaining the relatively high frequency of medical consultations among the elderly population. This multifaceted scenario underscores the challenges posed by shifting demographics and the need to address the distinct vulnerabilities of various population segments when devising strategies for mitigating and/or responding to disaster.

Regarding children’s consultations, the number of pediatric consultations decreased following the subacute phase. This may reflect that the introduction of mobile pharmacy in the hyperacute phase have suppressed the symptoms of children [16]. Furthermore, the gradual shift of pediatric consultations from shelters to local medical facilities, as well as those of adults and the elderly, may have contributed to the decrease in the number of patients. According to reports of Hitoyoshi Medical Center, which was the main disaster base hospital in the disaster area, two days after the disaster (July 6, 2020), Hitoyoshi Medical Center reopened; four days later (July 8, 2020), 27 of the total 45 hospitals affiliated with Hitoyoshi Medical Center reopened; six days later (July 10, 2020), 31 hospitals reopened; 16 days later (July 20, 2020), 34 hospitals reopened; and 26 days later (July 30, 2020), 38 hospitals reopened [22].

Pregnant women seeking medical consultations in the current study and previous study of other disaster made up a low proportion of all consultations (1.2%, 1.5% respectively) [23, 24]. One previous report found that pregnant women of the studied population tended to avoid evacuating to crowded shelters with limited personal space, instead tending to stay in their own cars [23]. The Kumamoto heavy rain occurred during the COVID-19 pandemic; pregnant women were likely to be even more cautious about evacuating to shelters due to COVID-19 infection risks. This might have limited their access to EMTs services, which were mainly provided at shelters during the disaster. Previous studies reported that the proportion of severe cases among pregnant women who visited the EMTs was significantly higher than that of other age groups [23, 25, 26]. This indicates that effective collaboration between EMTs and local health personnel, including midwives and public health nurses, is needed to ensure that pregnant women have appropriate access to clinical services during a disaster. This collaborative effort should seek to ensure that pregnant women receive comprehensive medical support, both within and beyond evacuation centers.

As a reason for consultation, skin diseases were more prevalent among children compared to adults and the elderly. This pattern aligns with findings from the 2018 West Japan heavy rain [7], and may be related to factors such as high temperature and humidity, hygiene constraints linked to limited water usage, bathing facilities in evacuation centers, exacerbation of atopic dermatitis due to psychological stress, and the emergence of food allergies resulting from restricted diets during evacuation [16, 27–30]. In the present study, the proportion of children presenting for disaster-related stress was lower than that observed in adults (2.4% versus 9.3%, respectively), which was consistent with the patterns seen during the West Japan heavy rain [7]. However, since children are less inclined to communicate their stress symptoms compared to adults [31], these results might underestimate the prevalence of disaster stress-related symptoms among children. Treatment interruptions, which were notably more prevalent during the hyperacute phase, can have serious clinical consequences. For example, discontinuation of drug treatments for allergic conditions(e.g., atopic dermatitis [32] and bronchial asthma) and disruptions to specialized dietary regimens during evacuation center residency are vital issues [28] that should be considered in future disaster preparedness and medical response strategies.

In considering ARIs, it is important to note that the COVID-19 pandemic had significantly impacted the incidence of ARIs during the disaster studied herein. Research comparing the incidence of ARIs before and during COVID-19 found that there was a substantial drop in the overall rate of total consultations (from 5.4 to 1.2%, respectively). This decline can be attributed to the rigorous implementation of infection-preventing measures during the COVID-19 pandemic [33]. However, we found that the prevalence of ARI consultations appeared to be relatively stable among pediatric patients: this rate was 12.8% during the West Japan heavy rain that occurred before COVID-19 [7] and 12.2% during the Kumamoto heavy rain that occurred during the COVID-19 pandemic. This is thought to reflect that children have a harder time adhering to preventive measures, such as maintaining social distancing and consistently wearing masks, in comparison to other age groups [33].

Among adults, wounds and minor trauma emerged as the prevailing health events and occurred predominantly during the acute phase rather than immediately following the disaster. This may reflect that adults were often injured while cleaning and repairing their homes in the wake of the disaster [34].

Regarding disaster-related stress, the number of consultations increased during the hyperacute phase, peaked in the subacute I phase, and decreased during the chronic phase. This pattern aligns with findings from other studies [35]. The increase in disaster-related stress could indicate that adults experienced stress stemming from damage to their homes coupled with the challenges encountered in the recovery process [36]. 

Among the elderly, the prevalence of hypertension peaked during the hyperacute phase. In disaster situations, environmental changes, stress, and sleep disruptions trigger sympathetic nerve activation; this leads to peripheral blood vessel constriction and heightened cardiac output, increasing blood pressure [32, 37]. Notably, treatment interruptions were frequently observed among the elderly during the hyperacute phase.

The percentage of treatment interruptions found herein was significantly higher (12.4%) than previously reported for the 2018 West Japan heavy rains and Kumamoto earthquakes 2016 [7, 9] (4.6% and 4.3%, respectively). This difference could reflect the distinct characteristics of the affected areas. The West Japan heavy rains affected a wide region, including places like Mabi-cho and Kurashiki City in Okayama, where the population was denser and medical facilities were more abundant. Conversely, the region impacted by the Kumamoto heavy rains was predominantly mountainous and inherently had fewer EMTs and medical institutions.

Regarding wounds, the incidence rate of 9.9% in the current study closely resembles that observed during the West Japan heavy rain (11.3%) [34]. Here, more wound cases were observed during the acute phase compared to the hyperacute phase. This trend may reflect that increases in wound cases were linked to increases in activities associated with cleaning and restoring homes in the aftermath of the disaster. Adults, in particular, are likely to engage in such activities during the acute phase, which could explain the higher number of wound cases during this phase.

These findings emphasize the importance of enacting injury-preventing measures and providing appropriate wound-care resources during the acute phase of a disaster response, when individuals are actively engaged in recovery efforts and may be at a higher risk of sustaining injuries.

This study, which represents the third paper in a series of descriptive analyses employing J-SPEED data, builds upon the groundwork laid by previous analyses of data collected during the Hokkaido Eastern Iburi Earthquake 2018 [38] and the West Japan heavy rain 2018 [7]. Notably, our current findings closely align with those of the study on the West Japan heavy rain 2018 in terms of the prevalence of EMT-managed health events, as discussed earlier and presented in detail in additional file 2. This alignment strongly implies that forthcoming studies within similar contexts may continue to support and complement the previous findings. This cumulative reinforcement of insights has the potential to significantly improve the ability of EMTs to prepare for and respond to disasters.

The findings of this study provide valuable insights that could be used to enhance the capabilities of EMTs in future disaster response efforts. A notable observation is that five major health events constituted a significant portion of the total consultations, with the hyperacute and acute phases accounting for 74% of these cases. Given the constraints on transporting medical supplies to disaster areas, EMTs could strategically bolster their resources to align with these prevalent health events during the immediate aftermath of a disaster. This could enable EMTs to more efficiently provide care to a large number of individuals in need.

Furthermore, as the proportion of these five major health events tended to decline gradually after the acute phase EMTs entering the disaster area after the subacute phase could make judicious use of the limited storage space within their vehicles and bags by carrying a selected drugs and equipment that cater to other health events. A practical strategy could involve optimizing the storage capacity of EMTs’ vehicles and bags by carefully curating a compact yet essential assortment of supplies and equipment for transport at different phases. This approach could ensure that the available space is utilized efficiently, which would facilitate the swift and effective deployment of resources while still addressing critical medical needs during disaster response operations.

Another crucial insight from this study is the need to address treatment interruptions during the hyperacute phase of a disaster, particularly for children and the elderly. In response to the Kumamoto heavy rain, the EMTs utilized the J-SPEED data to promptly identify and address the issue of treatment interruption just 2 days after their deployment. Subsequently, mobile pharmacies were dispatched to tackle this challenge [16].

Given that EMTs are limited in the amounts and types of medicines they can carry, the results of J-SPEED data analysis could be used to strengthen the early dispatch system for mobile pharmacies that provide a more diverse range of essential medications. This would facilitate the fulfillment of a wider spectrum of medication requirements during a disaster response.

The study found that the proportions of events directly or indirectly related to the disaster decreased gradually over time, while events unrelated to the disaster gradually increased. This trend aligns with earlier findings [7]. In the light of such observations, the data-driven decision was made to withdraw EMTs on day 22 of the disaster response (the chronic II phase) [16]. As disaster relevance is not confined to individual health events, but rather serves as a comprehensive metric, these findings underscore the importance of employing pragmatic indicators to guide medical operations during a disaster. The ongoing utilization of J-SPEED data holds the promise to help decision makers better understand disaster relevance, track changes in re-visit rates, and assess the number of necessary medical follow-ups.

## Limitations

This study does have certain limitations that should be acknowledged. Firstly, the analyzed data were exclusively those reported by EMTs; this study did not include data collected at certain medical institutions that were involved in providing healthcare during the disaster response. This omission could potentially lead to some significant events being overlooked, hindering the ability of this analysis to provide a comprehensive understanding of the overall impact on victims. Even given this, however, the study’s findings still provide valuable insights that can be used to strengthen the EMTs’ operation system. Secondly, since EMT members are busy during a disaster response, J-SPEED data could potentially suffer from underreporting due to the provision of incomplete data. Despite this, however, the data are likely to provide a comprehensive overview of the typical scenarios in which specific gender and age groups require medical assistance for particular health events within similar contexts.

## Conclusions

From a comprehensive analysis of health data collected using J-SPEED during the Kumamoto heavy rain, several key insights emerged. First, children commonly experienced skin diseases, treatment interruptions, and ARIs; adults were more prone to wounds, minor traumas, and stress; and the elderly more commonly presented with hypertension, treatment interruption, and wounds. A significant finding is that five major health events collectively represented half of all cases, suggesting the potential for EMTs to perform efficient, evidence-based resource allocation. Notably, treatment interruptions were prominent during the hyperacute phase across all age groups, emphasizing the need for a strong early response system. The metric “relatedness to disaster” proved to be useful as a practical indicator for comprehensively grasping the relation to disasters. By harnessing data from J-SPEED, which serves as a standardized reporting system for EMTs, this research demonstrates the feasibility of collecting, quantifying, and analyzing data using a uniform format. The study’s success in conducting comparative analyses with J-SPEED-based reports from previous disasters that varied in time, location, and/or disaster type showcases the potential to leverage accumulated understanding of past experiences to advance knowledge on disaster medicine and disaster public health. Ultimately, this research, alongside other disaster-related studies, holds the promise to enhance the quality of medical care and strengthen medical coordination efforts in future disaster responses.

### Electronic supplementary material

Below is the link to the electronic supplementary material.

Supplementary Material 1

Supplementary Material 1

## Data Availability

Restrictions apply to the availability of these data. Data was obtained from the J-SPEED Research Group for research purpose and are available with the permission of the group.

## Abbreviations

ARI: Acute Respiratory Infection
DPC: Daily Percentage Change
EMT: Emergency Medical Team
EMTCC: EMT Coordination Cell
EMTCC: Japan-Surveillance in Post Extreme Emergencies and Disasters
JSPS KAKENHI: Japan Society for the Promotion of Science, Grants-in-Aid for Scientific Research
MDS: Minimum Data Set
