# Supplementary figures and images for: Medical needs during the Kumamoto heavy rain 2020: analysis from emergency medical teams’ responses
Source: BMC Emerg Med. 2024 May 31;24:94. doi: 10.1186/s12873-024-01009-7 (PMC11141056; doi:10.1186/s12873-024-01009-7)

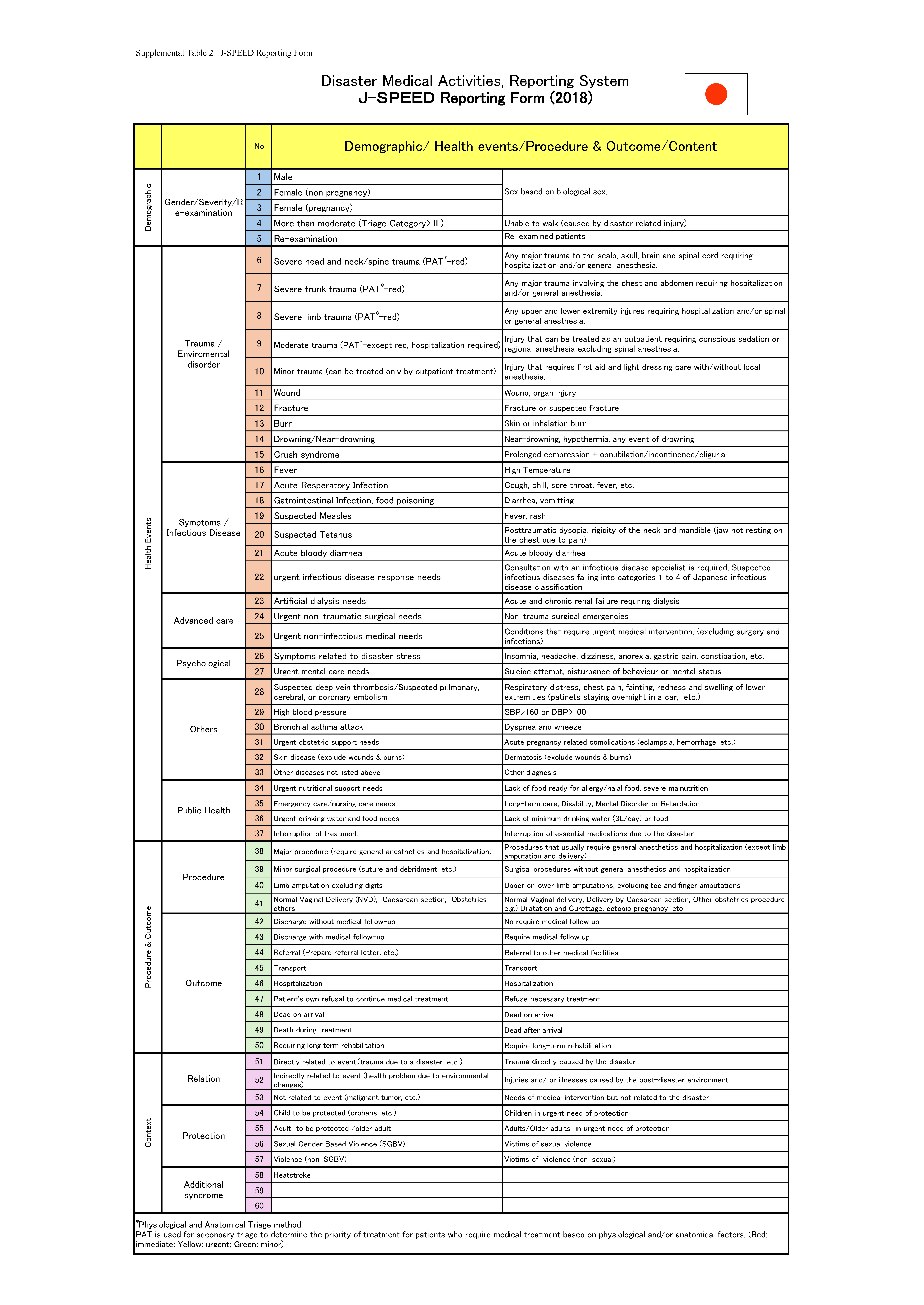

Supplement: Supplementary file 1 — Supplementary Material 1 [file 12873_2024_1009_MOESM1_ESM.tif]

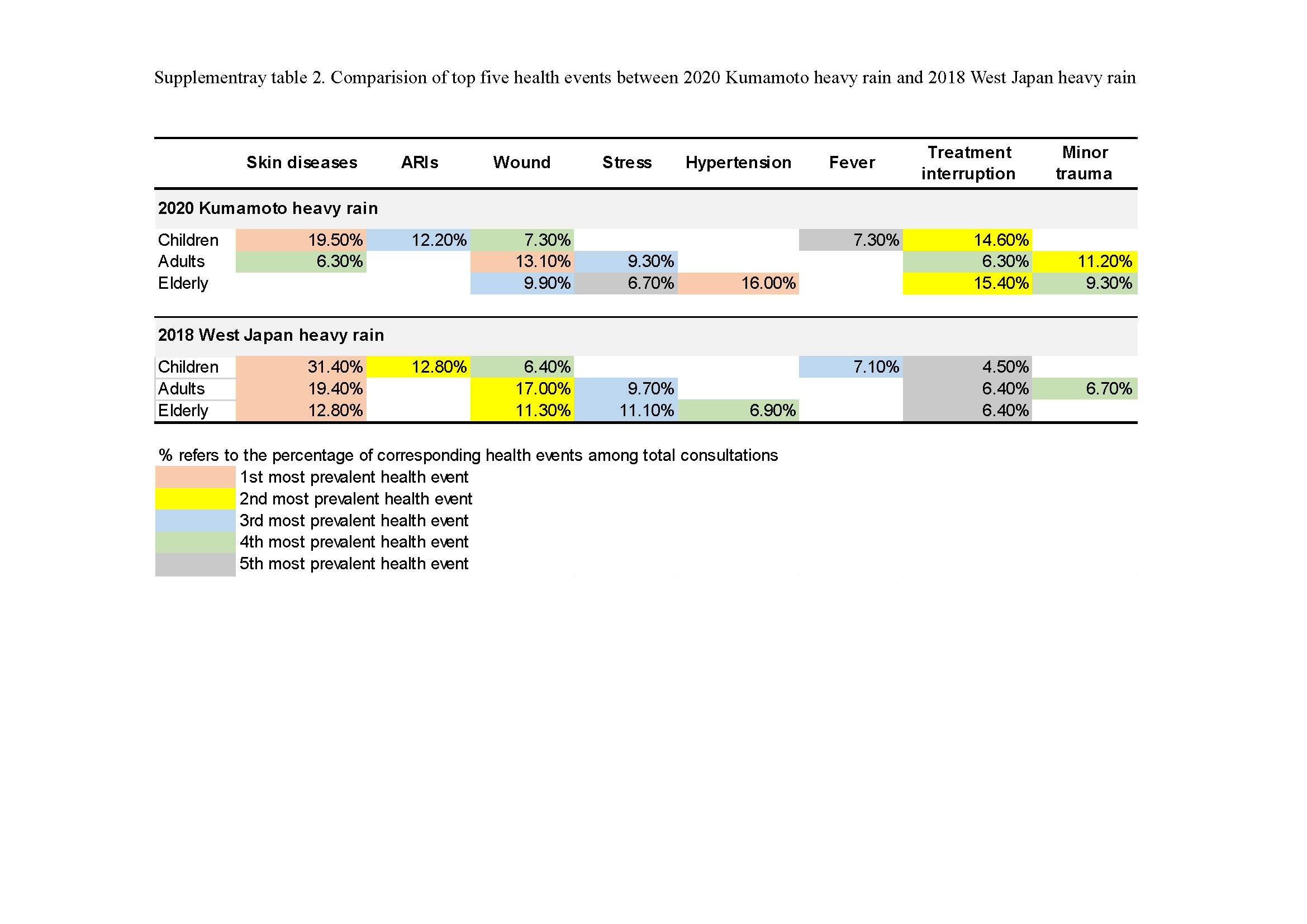

Supplement: Supplementary file 2 — Supplementary Material 1 [file 12873_2024_1009_MOESM2_ESM.tiff]
